# Transmission and Age Impact the Risk of Developing Febrile Malaria in Children with Asymptomatic *Plasmodium falciparum* Parasitemia

**DOI:** 10.1093/infdis/jiy591

**Published:** 2018-10-11

**Authors:** Kevin Wamae, Juliana Wambua, George Nyangweso, Gabriel Mwambingu, Faith Osier, Francis Ndung’u, Philip Bejon, Lynette Isabella Ochola-Oyier

**Affiliations:** 1 Kenya Medical Research Institute Wellcome Trust Research Programme, Kilifi, Kenya; 2 Nuffield Department of Medicine, Centre for Clinical Vaccinology and Tropical Medicine, Churchill Hospital, University of Oxford, United Kingdom; 3 Centre for Biotechnology and Bioinformatics, University of Nairobi, Kenya

**Keywords:** *Plasmodium falciparum*, asymptomatic, age, transmission, immunity

## Abstract

**Background:**

*Plasmodium falciparum* infections lead to febrile illness unless the host has sufficient immunity, in which case infection may cause no immediate symptoms (ie, “asymptomatic parasitemia”). Previous studies are conflicting on the role of asymptomatic parasitemia in determining the risk of developing febrile malaria.

**Methods:**

We monitored 2513 children (living in Kilifi, Kenyan Coast) by blood smears in 17 cross-sectional surveys to identify asymptomatic parasitemia and used active surveillance over 11325 child-years of follow-up to detect febrile malaria. We evaluated the interaction between transmission intensity, age, and asymptomatic parasitemia in determining the risk of developing febrile malaria.

**Results:**

In the moderate and high transmission intensity settings, asymptomatic parasitemia was associated with a reduced risk of febrile malaria in older children (> 3 years), while in the lower transmission setting, asymptomatic parasitemia was associated with an increased risk of febrile malaria in children of all ages. Additionally, the risk associated with asymptomatic parasitemia was limited to the first 90 days of follow-up.

**Conclusions:**

Asymptomatic parasitemia is modified by transmission intensity and age, altering the risk of developing febrile episodes and suggesting that host immunity plays a prominent role in mediating this process.

## Teaser

We present data from 3 cohorts in 1 geographical area with varying levels of malaria transmission. The results show that asymptomatic parasitemia is modified by transmission level and age, which alter the risk of developing febrile malaria.

In 2016, there were an estimated 216 million new malaria cases and approximately 445000 deaths globally, making malaria a significant health problem [1]. *Plasmodium falciparum* infections are characterized by disease manifestations that range from asymptomatic infection, mild febrile episodes, through to severe malaria. Asymptomatic parasitemia is widespread, affecting 24% of the population in sub-Saharan Africa [2], and might indicate that the host is at risk of developing febrile malaria at a later date (either with the original infecting parasites or due to an increased risk of superinfection), or that the host is immune and is therefore at a reduced risk of febrile malaria. Although the term “asymptomatic parasitemia” implies that there is no clinical consequence, studies have associated chronic asymptomatic infection with elevation of C-reactive protein (a biomarker of inflammation) [3, 4], lower platelet counts and hemoglobin levels, as well as higher levels of von Willebrand factor and platelet factor-4 (markers of endothelial and platelet activation, respectively) [3], low birth weight and premature births in pregnancy [5], cognitive impairment [6], malnutrition [7], and anemia [7–9]. These data have led to the proposal that asymptomatic parasitemia could be termed “chronic malaria” [10].

There are reports that asymptomatic infections are associated with reduced febrile malaria in infected individuals. Premunition is one mechanism by which individuals with asymptomatic parasitemia have been proposed to develop partial immunity to superinfection and resulting febrile malaria by long-term asymptomatic carriage of parasites [11, 12]. In the case of premunition, one would expect that clearing asymptomatic parasitemia would then make these individuals more susceptible to febrile malaria. However, there are conflicting reports, with some studies revealing that treatment of asymptomatic parasitemia was associated with increased risk of febrile malaria [13–18] while other studies found no effect [19, 20]. Therefore, there is still a gap in our understanding of the role of immunity in the maintenance of parasites in asymptomatic individuals.

Individuals living in high malaria transmission settings have been shown to have higher levels of immunity [21, 22] compared to those in low transmission settings. Therefore, understanding the risk of developing febrile malaria in asymptomatic individuals living in different transmission settings may help provide insights into the impact of immunity.

Some previous studies have found that asymptomatic parasitemia is associated with increased risk but other studies found a reduced risk of subsequent febrile malaria. In a study of 316 children aged 6 months to 5 years in an area of moderate malaria transmission in Uganda, children with asymptomatic parasitemia were at a higher risk of febrile malaria compared to the uninfected children within the first 30 days of detection [23]. Likewise, in a study of 566 children aged 2 to 17 years in an area of low malaria transmission in Senegal, it was shown that asymptomatic parasitemia at the beginning of the transmission season was associated with increased febrile malaria episodes, independent of age. However, this association was not observed at the end of the transmission season [24]. In contrast, in a study of 610 children under 6 years of age in an area of high malaria transmission in Tanzania, younger children (<3 years of age) with asymptomatic parasitemia at baseline were observed to be at a higher risk of febrile malaria while older children (>3 years of age) appeared to be at a reduced risk [25]. Similarly, in a larger study of 1356 children aged 2 to 18 years in an area of high malaria transmission in Senegal, it was shown that children with asymptomatic parasitemia in the dry season leading into the rainy season were at a lower risk of developing febrile malaria. Additionally, this association was age dependent as younger children (≤5 years of age) were found to be at a higher risk than older children (>5 years of age) [26]. Finally, in a study of 695 individuals aged 6 months to 25 years in an area of high malaria transmission in Mali with asymptomatic parasitemia at the end of the dry season were at a lower risk of febrile malaria in the subsequent malaria transmission season. The study also found that older individuals experienced less febrile malaria than younger children [20].

Overall, in the high transmission settings there was a reduced risk of developing febrile malaria and this could be stratified by age, while the moderate transmission settings showed an increased risk of developing febrile malaria. However, the limitations of these studies are that they were conducted using differing methodologies and therefore conclusions based on the assumption that differences in transmission intensity and age explain the differing results require confirmation.

We therefore sought to evaluate the role of malaria transmission intensity and age as potential effect modifiers on the risk of children with asymptomatic parasitemia developing febrile malaria, using data from 3 cohorts of children in different transmission intensities in Kilifi, Kenya.

## METHODS

### Study Design and Data Collection

The studies presented here were covered under the Kenya Medical Research Institute Scientific Ethics Research Unit protocol number 3149. The data used for this analysis were based on 3 cohorts in Kilifi of varying malaria transmission intensities, comprising Ngerenya (low transmission), Junju (moderate to high transmission), and Chonyi (high transmission). The data were prospectively collected between 1998 and 2014 for Ngerenya, 2005 and 2010 for Junju, and 1999 and 2001 for Chonyi. In these cohorts children were recruited at birth for weekly clinical malaria monitoring until the age of 15 years [27].

Although the transmission intensity was on a general decline during the study period [28, 29], it was much higher in Junju and Chonyi than in Ngerenya. We used data from the active weekly surveillance to determine malaria episodes and annual cross-sectional surveys to define asymptomatic parasitemia and uninfected children. There are 2 rainy seasons per year in Kenya, during which malaria transmission increases. The cross-sectional surveys are conducted before the long rains that occur in May to July, with the short rains occurring in October to November [30].

### Case Definitions

We defined asymptomatic parasitemia as having any detectable *P. falciparum* parasites by microscopy (thick and thin smears) and having an axillary temperature less than 37.5°C and no history of fever during the cross-sectional survey. Additionally, the children should not have had a recent febrile malaria episode within the month prior to the survey or had a fever within the subsequent 7 days from the date of the survey. Uninfected children were defined as those without *P. falciparum* parasitemia and no fever during the cross-sectional survey.

A febrile malaria episode was defined as having ≥ 2500 parasites/µL and an axillary temperature greater than 37.5°C. This definition is based on parasite cutoffs previously defined for these cohorts [30]; however, in order to avoid bias in definitions when analyzing the impact of interactions between asymptomatic infections and age and/or malaria transmission intensity, we adopted a single parasite density cutoff of ≥ 2500 parasites/µL for all age groups and all cohorts.

### Statistical Analysis

Survival analysis was used to compare the risk of developing febrile malaria between the asymptomatic and uninfected children and survival times were estimated as follows: the baseline (time 0) was defined as the date of cross-sectional survey while the end of follow-up was set at the last visit or at 365 days if follow-up continued to the subsequent annual cross-sectional survey; the time to first febrile malaria episode was defined as the interval between the baseline date and the first febrile malaria episode. Children who did not complete follow-up or did not get a febrile episode by the end of follow-up were censored.

We used Kaplan-Meier curves and log-rank test to compare time to first febrile malaria episode and the Cox proportional hazards model using transmission intensity and asymptomatic parasitemia as categorical variables, and age as a continuous linear and nonlinear variable (after transformation using multiple fractional polynomials in multivariable models). We adjusted for calendar year as a continuous variable to account for falling transmission intensity and the nonindependence of repeated observations using the robust cluster Huber-White method. We tested the proportional hazards assumption using Schoenfeld residuals and included interactions with time for the covariates that showed nonproportional hazards.

Data cleaning, plotting of Kaplan-Meier curves, and log-rank tests were carried out in R v3.5.1 [31] using the following R packages: data.Table v1.11.4 [32], dplyr v0.7.6 [33], survival v2.42–6 [34], and survminer v0.4.3 [35]. Cox regression analysis was performed in Stata v14.0.

## RESULTS

### Demography

**Figure 1.**
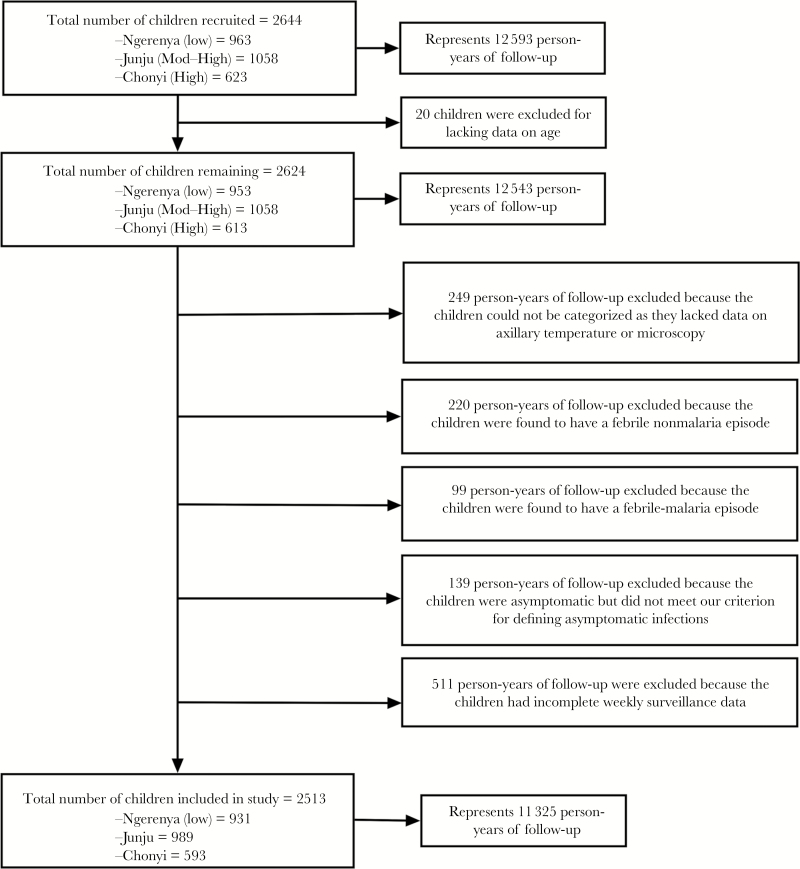
A flow chart showing the total number of children recruited in the cohorts and the numbers of children that met the case definitions for this study.

A total of 2644 children were recruited into the study over 17 cross-sectional surveys. Twenty children were excluded due to missing data on age, bringing the person-years of follow-up to 12543. A total of 1482 person-years of follow-up were excluded from the cross-sectional surveys for the following reasons: 249 lacked data on parasitemia or temperature, 220 had a febrile nonmalaria episode, 99 had febrile malaria, 139 asymptomatics who did not meet our criteria for defining an asymptomatic infection (see Methods), and 511 lacked weekly surveillance data. Consequently, we analyzed data on 2513 children, representing 11325 person-years of follow-up (Figure 1). There was a total of 3132 episodes of malaria while at the point of cross-sectional survey; 15.15% of children had asymptomatic parasitemia across all sites (Table 1).

**Table 1.** Demographic Characteristics of Study Participants

	Cohort		
	Low Transmission (Ngerenya)	Moderate–High Transmission (Junju)	High Transmission (Chonyi)
Total number of children included in the study	931	989	593
Age range, years	>0–15	>0–15	>0–15
Person years of follow-up	5179	5819	1595
Total number of males (%)	484 (51.99)	497 (50.25)	306 (51.60)
Total number asymptomatic episodes (%)	419 (9.50)	923 (17.00)	538 (36.40)
Total number children with 1 or more febrile malaria episodes (%)	162 (17.40)	371 (37.51)	119 (20.06)

### Risk of Developing Febrile Malaria

**Figure 2.**
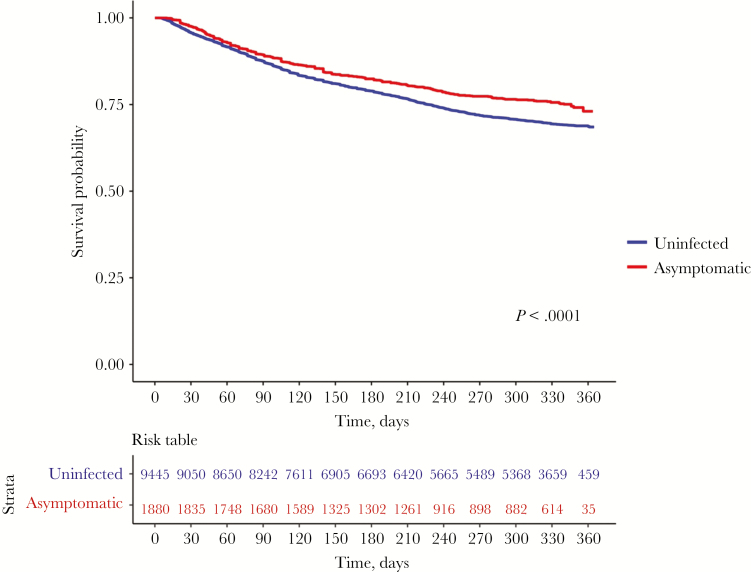
Risk of developing febrile malaria in uninfected children versus children with asymptomatic infections across the 3 malaria transmission settings. This plot compares the time to first febrile malaria episode between uninfected children versus children with asymptomatic infections across the different malaria transmission settings. The risk table shows the number of participants under observation at every 30-days interval for both the uninfected (blue) and asymptomatic (red) groups. The log-rank test was used compare the survival distributions between the 2 groups (*P* < .0001).

We compared the risk of developing febrile malaria between children with asymptomatic parasitemia and uninfected children at baseline. Uninfected children were at a higher risk compared to children with asymptomatic infections from the start of follow-up. This difference in rates of developing episodes was more distinct after 90 days, when the 2 survival functions diverged to the end of follow-up (Figure 2; *P* value <.0001).

**Figure 3.**
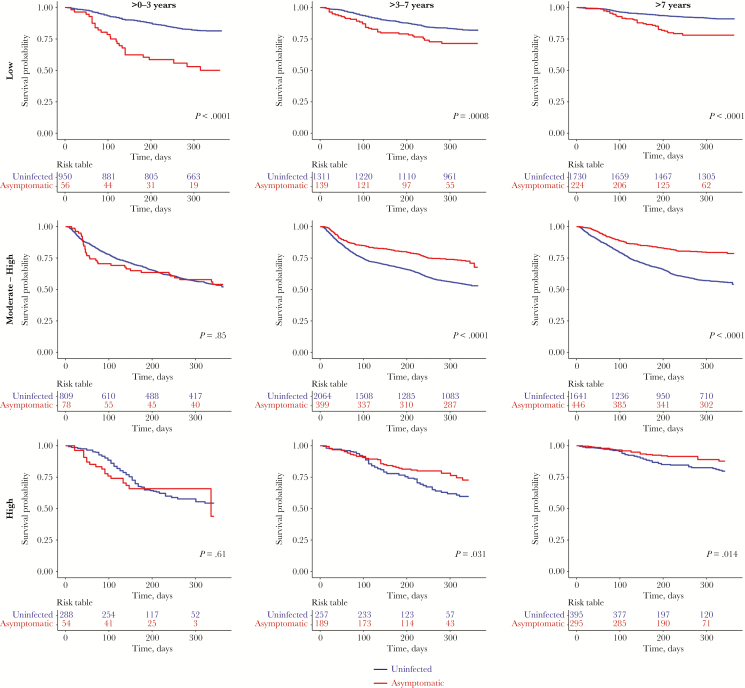
Risk of developing febrile malaria between asymptomatic and uninfected children, stratified by malaria transmission intensity and age. The plots compare the time to first febrile malaria episode between the uninfected children versus children with asymptomatic infections across the different malaria transmission settings, stratified by 3 age groups: 0–3, >3–7 and >7 years old. The risk table shows the number of participants under observation at every 100-day interval for both the uninfected (blue) and asymptomatic (red) groups. The log-rank test was used compare the survival distributions.

We then examined the effect of age and transmission intensity on the risk of developing febrile malaria in children with asymptomatic parasitemia versus uninfected children. Asymptomatic parasitemia was associated with an increased risk of febrile malaria within all age groups in the low transmission setting. The risk of febrile malaria was not altered by asymptomatic parasitemia among the younger age groups (≤ 3 years) in the moderate to high and in the high transmission settings. However, among the older age groups (> 3 years) in the moderate-high and high transmission settings, asymptomatic parasitemia was associated with a reduced risk of developing febrile malaria (Figure 3).

There were some variations in effect over time. The risk among children with asymptomatic infection decreased over time relative to the risk among uninfected children. In settings where asymptomatic infection was associated with increased risk (ie, young children and lower transmission) the survival functions of asymptomatic children compared to uninfected children diverged before 90 days, and then became parallel after 90 days. This contrasts with settings where asymptomatic parasitemia was associated with reduced risk (ie, older children and higher transmission), in which the survival functions of asymptomatic and uninfected individuals were similar before 90 days, and then showed an increased risk among children with asymptomatic parasitemia.

### The Impact of Transmission Intensity and Age on Risk of Developing Febrile Malaria

We constructed a Cox regression model that tested the effect of transmission intensity, infection status (uninfected vs asymptomatic), transformed age, sex (male vs female), year of survey and time cutoffs (days where the 2 survival functions diverge), and their interactions. Age as a nonlinear transformation, using the multiple fractional polynomial approach, was found to be a better fit than a linear variable (*P* < .0001; Supplementary Figure 1). Therefore, using age as a nonlinear variable, we found interactions at baseline to be statistically significant between asymptomatic parasitemia and transmission intensity (*P* < .0001), between transformed age and transmission intensity (*P* < .0001), between asymptomatic parasitemia and transformed age (*P* < .0001), as well as between asymptomatic parasitemia and year of survey (*P* < .0001). The variations in the effects of infection status and year of survey over time were statistically significant (see Schoenfeld residuals, Supplementary Table 1). We then examined models that allowed linear and logarithmic interactions between time and variables (transmission, transformed age, infection status, and year of survey), as well as step-wise interactions between time and variables at cutoffs of 30, 60, 90, and 120 days. We found the model with a cutoff at 90 days to be a better fit of the data based on log-likelihood, Akaike information criterion, and Bayesian information criterion scores (Supplementary Table 10). Additionally, an exploratory analysis showed that every 10-fold increase in asymptomatic parasite density was associated with a 37% increased risk of developing a febrile malaria (Supplementary Table 2).

Finally, we conducted a sensitivity analysis using different parasite density thresholds for defining febrile malaria as per previous work (ie, any parasitemia in children <1 year and ≥ 2500 parasites/μL for older children) to evaluate risk [30]. The effect of asymptomatic parasitemia remained the same (Supplementary Table 11) as when we used 1 cutoff to define febrile malaria (ie, ≥ 2500 parasites/μL for all children). The detailed process of model optimization is shown in Supplementary Table 3–9 and the comparison of goodness-of-fit statistics in Supplementary Table 10. The final retained model is shown in Table 2.

**Table 2.** Multivariable Analysis to Test the Effect of Different Covariates on the Risk of Developing Febrile Malaria

Covariate	Hazard Ratio	Robust SE	z	*P*>|z|	Confidence Interval	
					Lower	Upper
Main						
Transmission (high vs low)	0.98	0.35	−0.05	.957	0.49	1.96
Transmission (high vs mod-high)	16.55	5.43	8.55	**<.0001**	8.70	31.49
Transformed age	5.29	1.50	5.86	**<.0001**	3.03	9.22
Transmission (high vs low) **×** transformed age	0.60	0.21	−1.43	.154	0.30	1.21
Transmission (high vs mod-high) **×** transformed age	0.24	0.07	−4.59	**<.0001**	0.13	0.44
Infection status (uninfected vs asymptomatic)	0.21	0.07	−4.97	**<.0001**	0.12	0.39
Transmission (high vs low) **×** infection status (uninfected vs asymptomatic)	2.43	0.45	4.81	**<.0001**	1.69	3.49
Transmission (high vs mod-high) **×** infection status (uninfected vs asymptomatic)	0.25	0.07	−5.29	**<.0001**	0.15	0.42
Infection status (uninfected vs asymptomatic) **×** transformed age	3.32	0.93	4.27	**<.0001**	1.91	5.76
Year of survey	0.95	0.01	−5.72	**<.0001**	0.93	0.97
Infection status (uninfected vs asymptomatic) **×** year of survey	1.12	0.02	5.35	**<.0001**	1.07	1.17
Time varying covariates						
Infection status (uninfected vs asymptomatic)	0.69	0.08	−3.39	**0.001**	0.56	0.85
Year of survey	0.94	0.01	−7.34	**<0.0001**	0.92	0.95

Abbreviations: mod-high, moderate-high; SE, standard error.

This final model shows the effect of the different covariates on developing febrile episodes with alternative cutoff times of <90 and >90 days. The *P* values in bold represent those that were statistically significant (*P* < .05). The symbol **×** indicates an interaction between the respective covariates.

## DISCUSSION

Asymptomatic parasitemia before the malaria transmission season predicted the risk of developing febrile malaria. This effect was significantly modified by transmission intensity and age. In the moderate to high and high transmission settings, asymptomatic parasitemia was associated with a reduced risk of febrile malaria in older children (>3–15 years). There was, however, no association between asymptomatic parasitemia and risk in younger children in the moderate to high and high transmission settings. In contrast, in children of all ages in the lower transmission setting, asymptomatic parasitemia was associated with an increased risk of febrile malaria. The effect of asymptomatic parasitemia was modified by time since ascertainment. At low transmission, asymptomatics were at a higher risk than uninfected children until 90 days after ascertainment, following which asymptomatics were at similar risk to uninfected children. On the other hand, at higher transmission and older age, asymptomatics were at a similar risk as uninfected children until 90 days after ascertainment, following which asymptomatics were at a lower risk than uninfected children.

This study is in agreement with studies conducted in high transmission areas where asymptomatic parasitemia has previously been associated with a reduced risk of febrile episodes [20, 25, 26], in particular among older children. Additionally, in low transmission areas some studies have shown that asymptomatic parasitemia was associated with an increased risk of febrile episodes [23, 24], again consistent with our findings. Thus, transmission intensity and age modified the effect of asymptomatic parasitemia on the risk of developing febrile malaria.

Children living in malaria endemic regions with high transmission acquire immunity at a faster rate than those living in low endemic regions, as they are repeatedly exposed to infections [22]. While immunity to malaria may not necessarily prevent infection, it may help an individual control parasite density and prevent symptoms, thus leading to asymptomatic parasitemia [22]. It has been argued that the presence of asymptomatic parasitemia leads to resistance to further infection, a state that is termed “premunition” [36]. Continuous exposure to malaria infection may continuously prime the immune system and lower the risk of developing febrile malaria. However, the presence of asymptomatic parasitemia implies a risk of parasites going on to cause febrile malaria and may also imply a higher degree of exposure to further infectious bites, and therefore may be associated with a higher risk of febrile malaria. In this study, we found that among older children at higher transmission intensity, having asymptomatic infections reduced the risk of developing febrile malaria, while younger children with a naive immune system were at a greater risk of developing febrile malaria if they had asymptomatic parasitemia. The reduced risk with asymptomatic parasitemia becomes more pronounced in older children, which is consistent with a prominent role for acquired immunity, albeit we cannot exclude an additional role for premunition. Previous studies have indicated that antibodies are predictive of reduced susceptibility to malaria among children with asymptomatic parasitemia but not among uninfected children [37, 38], implying a role for antibodies in mediating the protection seen in older children with asymptomatic parasitemia.

The 90-day period of increased risk following the ascertainment of asymptomatic parasitemia suggests there is a period during which parasitemia may lead to febrile malaria, but thereafter the likelihood of the parasite escaping immune control is reduced, hence also reducing the risk of febrile infections. Alternatively, the increased risk of febrile episodes may reflect increased exposure associated with the asymptomatic parasitemia [39] or exposure due to the rainy season, which indicates the malaria season. Chronic asymptomatic parasitemia is not thought to frequently cause subsequent febrile disease, because febrile malaria is highly seasonal, even in areas where asymptomatic parasitemia is highly prevalent [40], and in deliberate infection undertaken in neurosyphilis treatment, fever was seen early in the time course of infection [41].

We found in our cohorts that every 10-fold increase in parasite density was associated with an increased risk of developing a febrile episode. We speculate that higher-density asymptomatic parasitemia indicates less host immunity and therefore a greater risk that parasites will evade immune control.

Limitations of this study include the fact that it was conducted in a single geographical area (ie, Kilifi County on the Kenyan Coast). However, we included 3 different sites at different transmission settings and our findings are in agreement with those from other studies [20, 23–26]. Our reliance on microscopy diagnosis may have missed out a considerable number of children with submicroscopic infections because microscopy is less sensitive than polymerase chain reaction [42]. Misclassification of low-density asymptomatic infections as parasite negative would tend to reduce the strength of associations seen; hence, our study may be an underestimate of the significance of asymptomatic infections. Malaria transmission has been shown to be locally heterogeneous [43] and hence describing a cohort as uniformly high or low transmission is a simplification, but is necessary in order to study effect modification across groups at different transmission intensities. Episodes of malaria could have been missed by our surveillance, either because the episode was self-limiting or treatment was obtained elsewhere. In mitigation, we used active surveillance, which has been shown to be more sensitive than passive surveillance [44], and assuming that surveillance is similarly incomplete for all children, we would not predict a bias arising from this limitation. We did not have data to account for host population dynamics such as short-term migration [45], and environmental factors leading to heterogeneity of risk within cohorts [43], and these may impact on how transmission intensity, age, and asymptomatic parasitemia influence the risk of developing febrile malaria.

Our study cannot be used to infer the effects of treating asymptomatic parasitemia. Randomized trials of intermittent presumptive treatment show benefits across a range of transmission intensities and ages [46], despite the variation in the effect of asymptomatic parasitemia seen across transmission intensity and age in our observational study. Our study was conducted to study the natural history of asymptomatic parasitemia rather than to make policy recommendations.

Additional studies are required to examine whether episodes of febrile malaria are associated with parasites of a similar genotype to those involved in the prior asymptomatic infection, or whether the increased risk applies to all parasites. The former would imply a specific risk of asymptomatic parasitemia limited to a specific infection episode, and the latter would imply a general increase in susceptibility not limited to any specific episode.

## Supplementary Data

Supplementary materials are available at *The Journal of Infectious Diseases* online. Consisting of data provided by the authors to benefit the reader, the posted materials are not copyedited and are the sole responsibility of the authors, so questions or comments should be addressed to the corresponding author.

jiy591_suppl_Supplementary_Table_01

jiy591_suppl_Supplementary_Table_02

jiy591_suppl_Supplementary_Table_03

jiy591_suppl_Supplementary_Table_04

jiy591_suppl_Supplementary_Table_05

jiy591_suppl_Supplementary_Table_06

jiy591_suppl_Supplementary_Table_07

jiy591_suppl_Supplementary_Table_08

jiy591_suppl_Supplementary_Table_09

jiy591_suppl_Supplementary_Table_10

jiy591_suppl_Supplementary_Table_11

jiy591_suppl_Supplementary_Material

jiy591_suppl_Supplementary_Figure

## Notes

Presented in part: Keynote Symposia Conference Malaria: From Innovation to Eradication, Speke Resort and Conference Centre, Kampala, Uganda, 19–23 February 2017.

***Author contributions***. K. W. was responsible for data cleaning, analysis, interpretation and production of the final manuscript. J. W. led and coordinated the data collection and participated in drafting of the manuscript. G. N. coordinated collection and management of the data, and participated in drafting of the manuscript. G. M. was involved in microscopic analysis of the samples. F. O. and F. N. contributed to data collection and provided input to the manuscript. P. B. and L. I. O.-O. conceived and coordinated the study, participated in its design, analyzed the data, and wrote the manuscript.

***Acknowledgment.*** The participants and their parents are thanked.

***Disclaimer.***  The funders had no role in study design, data collection, analysis, interpretation, or report writing. The authors had full access to all data in the study and final responsibility for the decision to submit for publication. The views expressed in this publication are those of the authors and not necessarily those of African Academy of Sciences (AAS), New Partnership for Africa’s Development Planning and Coordinating (NEPAD) Agency, Wellcome Trust, or the UK government. This manuscript is published with the permission of the Director of Kenya Medical Research Institute.

***Financial support.*** This work was supported by the Developing Excellence in Leadership, Training and Science Africa Initiative (grant number DEL-15-003), an independent funding scheme of the AAS Alliance for Accelerating Excellence in Science in Africa and supported by the NEPAD Agency, with funding from the Wellcome Trust (grant number 107769/Z/10/Z) and the UK Government; and support from a Wellcome Trust Intermediate Fellowship (grant number 107568/Z/15/Z awarded to L.I.O-O).

***Potential conflicts of interest.*** All authors: No reported conflicts. All authors have submitted the ICMJE Form for Disclosure of Potential Conflicts of Interest. Conflicts that the editors consider relevant to the content of the manuscript have been disclosed.
