# Supplementary material for: Transmission and Age Impact the Risk of Developing Febrile Malaria in Children with Asymptomatic Plasmodium falciparum Parasitemia
Source: J Infect Dis. 2018 Oct 11;219(6):936–44. doi: 10.1093/infdis/jiy591 (PMC6386809; doi:10.1093/infdis/jiy591)
Supplement: jiy591_suppl_Supplementary_Table_01 [file jiy591_suppl_supplementary_table_01.docx]

**Supplementary Table 1. Schoenfeld’s residuals for the tested covariates.**

| **Co-variate** | **rho** | **chi2** | **df** | **Prob>chi2** |
| --- | --- | --- | --- | --- |
| Infection Status (Uninfected vs. Asymptomatic) | -0.041 | 7.26 | 1 | **0.007** |
| Transmission (High vs. Low) | -2E-04 | <0.01 | 1 | 0.99 |
| Transmission (High vs. Mod-High) | -0.024 | 2.54 | 1 | 0.11 |
| Age | -0.003 | 0.04 | 1 | 0.85 |
| Sex (Male vs. Female) | -0.008 | 0.42 | 1 | 0.52 |
| Year of Survey | -0.058 | 18.26 | 1 | **<0.0001** |
| Global Test |  | 95.61 | 6 | **<0.0001** |

The global p-value shows that the effect of at least one of the covariates varies over time. Looking at each of the covariates independently, the effects of infection status, age and year of survey vary over time as they have p-values that are below 0.05. Abbreviations: mod-high - moderate-high.
