# Supplementary material for: Transmission and Age Impact the Risk of Developing Febrile Malaria in Children with Asymptomatic Plasmodium falciparum Parasitemia
Source: J Infect Dis. 2018 Oct 11;219(6):936–44. doi: 10.1093/infdis/jiy591 (PMC6386809; doi:10.1093/infdis/jiy591)
Supplement: jiy591_suppl_Supplementary_Table_02 [file jiy591_suppl_supplementary_table_02.docx]

**Supplementary Table 2. Univariable analysis to test the effect of increasing asymptomatic parasitemia on the risk of developing febrile malaria.**

| **Covariate** | **Hazard Ratio** | **Std. Error** | **z** | **P>\|z\|** | **Confidence Interval** | | | |
| --- | --- | --- | --- | --- | --- | --- | --- | --- |
|  |  |  |  |  | **Lower** | | **Upper** | |
| Pf. Density* | 1.35 | 0.09 | 4.46 | **<0.0001** | 1.18 | 1.54 | |  |

The p-values in bold represent those that were statistically significant (p < 0.05).
