# Supplementary material for: Transmission and Age Impact the Risk of Developing Febrile Malaria in Children with Asymptomatic Plasmodium falciparum Parasitemia
Source: J Infect Dis. 2018 Oct 11;219(6):936–44. doi: 10.1093/infdis/jiy591 (PMC6386809; doi:10.1093/infdis/jiy591)
Supplement: jiy591_suppl_Supplementary_Table_03 [file jiy591_suppl_supplementary_table_03.docx]

**Supplementary Table 3. Multivariable analysis to test the effect of different covariates on the risk of developing febrile malaria**

| **Covariate** | **Hazard Ratio** | **Robust Std. Error** | **z** | **P>\|z\|** | **Confidence Interval** | |
| --- | --- | --- | --- | --- | --- | --- |
|  |  |  |  |  | **Lower** | **Upper** |
| Transmission (High vs. Low) | 0.92 | 0.32 | -0.23 | 0.815 | 0.47 | 1.82 |
| Transmission (High vs. Mod-High) | 15.92 | 5.16 | 8.54 | **<0.0001** | 8.44 | 30.06 |
| Transformed Age | 5.06 | 1.40 | 5.86 | **<0.0001** | 2.94 | 8.71 |
| Transmission (High vs. Low) **x** Transformed Age | 0.65 | 0.23 | -1.24 | 0.213 | 0.33 | 1.28 |
| Transmission (High vs. Mod-High) **x** Transformed Age | 0.25 | 0.08 | -4.58 | **<0.0001** | 0.13 | 0.45 |
| Infection Status (Uninfected vs. Asymptomatic) | 0.16 | 0.05 | -5.75 | **<0.0001** | 0.09 | 0.30 |
| Transmission (High vs. Low) **x** Infection Status (Uninfected vs. Asymptomatic) | 2.38 | 0.44 | 4.74 | **<0.0001** | 1.66 | 3.41 |
| Transmission (High vs. Mod-High) **x** Infection Status (Uninfected vs. Asymptomatic) | 0.24 | 0.06 | -5.46 | **<0.0001** | 0.15 | 0.40 |
| Infection Status (Uninfected vs. Asymptomatic) **x** Transformed Age | 3.44 | 0.98 | 4.35 | **<0.0001** | 1.97 | 6.02 |
| Sex (Male vs. Female) | 1.06 | 0.05 | 1.16 | 0.246 | 0.96 | 1.16 |
| Year of Survey | 0.92 | 0.01 | -10.25 | **<0.0001** | 0.90 | 0.93 |
| Infection Status (Uninfected vs. Asymptomatic) **x** Year of Survey | 1.13 | 0.02 | 5.6 | **<0.0001** | 1.08 | 1.18 |

The odds ratio at baseline for all the covariates are presented. Time was included as exponential. The p-values in bold represent those that were statistically significant (p < 0.05). The symbol **x** indicates an interaction between the respective covariates. Abbreviations: mod-high - moderate-high.
