# Supplementary material for: Transmission and Age Impact the Risk of Developing Febrile Malaria in Children with Asymptomatic Plasmodium falciparum Parasitemia
Source: J Infect Dis. 2018 Oct 11;219(6):936–44. doi: 10.1093/infdis/jiy591 (PMC6386809; doi:10.1093/infdis/jiy591)
Supplement: jiy591_suppl_Supplementary_Table_04 [file jiy591_suppl_supplementary_table_04.docx]

**Supplementary Table 4. Multivariable analysis to test the effect of different covariates on the risk of developing febrile malaria**

| **Covariate** | **Hazard Ratio** | **Robust Std. Error** | **z** | **P>\|z\|** | **Confidence Interval** | |
| --- | --- | --- | --- | --- | --- | --- |
|  |  |  |  |  | **Lower** | **Upper** |
| **Main** | | | | | | |
| Transmission (High vs. Low) | 0.99 | 0.35 | -0.04 | 0.972 | 0.49 | 1.98 |
| Transmission (High vs. Mod-High) | 16.69 | 5.49 | 8.56 | **<0.0001** | 8.76 | 31.80 |
| Transformed Age | 5.37 | 1.53 | 5.90 | **<0.0001** | 3.07 | 9.38 |
| Transmission (High vs. Low) **x** Transformed Age | 0.59 | 0.21 | -1.51 | 0.132 | 0.29 | 1.18 |
| Transmission (High vs. Mod-High) **x** Transformed Age | 0.23 | 0.07 | -4.67 | **<0.0001** | 0.13 | 0.43 |
| Infection Status (Uninfected vs. Asymptomatic) | 0.21 | 0.07 | -4.96 | **<0.0001** | 0.11 | 0.39 |
| Transmission (High vs. Low) **x** Infection Status (Uninfected vs. Asymptomatic) | 2.47 | 0.46 | 4.89 | **<0.0001** | 1.72 | 3.56 |
| Transmission (High vs. Mod-High) **x** Infection Status (Uninfected vs. Asymptomatic) | 0.26 | 0.07 | -5.23 | **<0.0001** | 0.15 | 0.43 |
| Infection Status (Uninfected vs. Asymptomatic) **x** Transformed Age | 3.33 | 0.94 | 4.25 | **<0.0001** | 1.91 | 5.78 |
| Sex (Male vs. Female) | 1.06 | 0.05 | 1.17 | 0.241 | 0.96 | 1.16 |
| Year of Survey | 0.95 | 0.01 | -4.74 | **<0.0001** | 0.94 | 0.97 |
| Infection Status (Uninfected vs. Asymptomatic) **x** Year of Survey | 1.12 | 0.02 | 5.46 | **<0.0001** | 1.08 | 1.17 |
| **Time Varying Covariates** | | | | | | |
| Infection Status (Uninfected vs. Asymptomatic) | 1.00 | 0.00 | -2.75 | 0.006 | 1.00 | 1.00 |
| Year | 1.00 | 0.00 | -6.83 | **<0.0001** | 1.00 | 1.00 |

The odds ratio at baseline (main) for all the covariates and those that varied over time (time varying covariates) are presented. Time was included as exponential. The p-values in bold represent those that were statistically significant (p < 0.05). The symbol **x** indicates an interaction between the respective covariates. Abbreviations: mod-high - moderate-high.
