# Supplementary material for: Transmission and Age Impact the Risk of Developing Febrile Malaria in Children with Asymptomatic Plasmodium falciparum Parasitemia
Source: J Infect Dis. 2018 Oct 11;219(6):936–44. doi: 10.1093/infdis/jiy591 (PMC6386809; doi:10.1093/infdis/jiy591)
Supplement: jiy591_suppl_Supplementary_Table_08 [file jiy591_suppl_supplementary_table_08.docx]

**Supplementary Table 8. Multivariable analysis to test the effect of different covariates on the risk of developing febrile malaria**

| **Covariate** | **Hazard Ratio** | **Robust Std. Error** | **z** | **P>\|z\|** | **Confidence Interval** | |  |
| --- | --- | --- | --- | --- | --- | --- | --- |
|  |  |  |  |  | **Lower** | **Upper** |  |
| **Main** | | | | | | | |
| Transmission (High vs. Low) | 0.96 | 0.34 | -0.11 | 0.912 | 0.48 | 1.92 |  |
| Transmission (High vs. Mod-High) | 16.37 | 5.35 | 8.55 | **<0.0001** | 8.63 | 31.07 |  |
| Transformed Age | 5.20 | 1.46 | 5.86 | **<0.0001** | 3.00 | 9.03 |  |
| Transmission (High vs. Low) **x** Transformed Age | 0.62 | 0.22 | -1.35 | 0.176 | 0.31 | 1.24 |  |
| Transmission (High vs. Mod-High) **x** Transformed Age | 0.24 | 0.08 | -4.57 | **<0.0001** | 0.13 | 0.44 |  |
| Infection Status (Uninfected vs. Asymptomatic) | 0.22 | 0.07 | -4.82 | **<0.0001** | 0.12 | 0.40 |  |
| Transmission (High vs. Low) **x** Infection Status (Uninfected vs. Asymptomatic) | 2.40 | 0.44 | 4.76 | **<0.0001** | 1.67 | 3.45 |  |
| Transmission (High vs. Mod-High) **x** Infection Status (Uninfected vs. Asymptomatic) | 0.25 | 0.06 | -5.38 | **<0.0001** | 0.15 | 0.41 |  |
| Infection Status (Uninfected vs. Asymptomatic) **x** Transformed Age | 3.35 | 0.94 | 4.29 | **<0.0001** | 1.93 | 5.82 |  |
| Year of Survey | 0.96 | 0.01 | -4.77 | **<0.0001** | 0.94 | 0.97 |  |
| Infection Status (Uninfected vs. Asymptomatic) **x** Year of Survey | 1.12 | 0.02 | 5.43 | **<0.0001** | 1.08 | 1.17 |  |
| **Time Varying Covariates** | | | | | | | |
| Infection Status (Uninfected vs. Asymptomatic) | 0.72 | 0.08 | -2.92 | **0.003** | 0.57 | 0.90 |  |
| Year of Survey | 0.94 | 0.01 | -6.50 | **<0.0001** | 0.92 | 0.96 |  |

The odds ratio at baseline (main) for tall the covariates and those that varied over time (time varying covariates) are presented. Also, sex was dropped as it was found not to be significant, time was included as exponential with alternative cut-offs of <60 and >60 days. The p-values in bold represent those that were statistically significant (p < 0.05). The symbol **x** indicates an interaction between the respective covariates. Abbreviations: mod-high - moderate-high.
