# Supplementary material for: Transmission and Age Impact the Risk of Developing Febrile Malaria in Children with Asymptomatic Plasmodium falciparum Parasitemia
Source: J Infect Dis. 2018 Oct 11;219(6):936–44. doi: 10.1093/infdis/jiy591 (PMC6386809; doi:10.1093/infdis/jiy591)
Supplement: jiy591_suppl_Supplementary_Table_10 [file jiy591_suppl_supplementary_table_10.docx]

**Supplementary Table 10. Markers of goodness-of-fit for the Cox-Regression Models**

| **Model** | **Reference** | **Log likelihood** | **df** | **AIC** | **BIC** |
| --- | --- | --- | --- | --- | --- |
| Multivariable model with a non-linear effect of age and its interactions, no tvc | Supplementary Table 3 | -27787.36 | 12 | 55598.72 | 55686.73 |
| Multivariable model with a non-linear effect of age and its interactions, with tvc | Supplementary Table 4 | -27756.43 | 14 | 55540.86 | 55643.54 |
| Multivariable model with time-varying covariates (statistically significant covariates) | Supplementary Table 5 | -27757.68 | 13 | 55541.36 | 55636.71 |
| Multivariable model with time-varying covariates (logarithmic variations with time) | Supplementary Table 6 | -27763.78 | 13 | 55553.55 | 55648.90 |
| Multivariable model with time-varying covariates (cut-offs) | 30-day cut-off (Supplementary Table 7) | -27782.10 | 13 | 55590.20 | 55685.55 |
|  | 60-day cut-off (Supplementary Table 8) | -27761.11 | 13 | 55548.23 | 55643.58 |
|  | 90- day cut-off (Table 2) | -27750.39 | 13 | 55526.79 | 55622.14 |
|  | 120- day cut-off (Supplementary Table 9) | -27770.23 | 13 | 55566.47 | 55661.82 |

The log-likelihood score is a statistical test used for comparing the goodness of fit of two models. Based on the likelihood ratio, it expresses how many times more likely the data are under one model than the other. Smaller log-likelihood values indicate a better model. Akaike's Information Criterion (AIC) and the Bayesian Information Criterion (BIC) are information-based criteria that assess model fit. In general, BIC penalizes models with more parameters more than AIC does. For this reason, it leads to choosing more parsimonious models, that is, models with fewer parameters, than does AIC. In both cases, lower AIC and BIC scores indicate a better model. Abbreviations: df – degrees of freedom, tvc – time varying covariates.
