# Supplementary material for: Transmission and Age Impact the Risk of Developing Febrile Malaria in Children with Asymptomatic Plasmodium falciparum Parasitemia
Source: J Infect Dis. 2018 Oct 11;219(6):936–44. doi: 10.1093/infdis/jiy591 (PMC6386809; doi:10.1093/infdis/jiy591)
Supplement: jiy591_suppl_Supplementary_Table_11 [file jiy591_suppl_supplementary_table_11.docx]

**Supplementary Table 11. Multivariable analysis to test the effect of different covariates on the risk of developing febrile malaria.**

| **Covariate** | **Hazard Ratio** | **Robust Std. Error** | **z** | **P>\|z\|** | **Confidence Interval** | |
| --- | --- | --- | --- | --- | --- | --- |
|  |  |  |  |  | **Lower** | **Upper** |
| **Main** | | | | | | |
| Transmission (High vs. Low) | 1.01 | 0.36 | 0.02 | 0.984 | 0.50 | 2.02 |
| Transmission (High vs. Mod-High) | 16.99 | 5.59 | 8.61 | **<0.0001** | 8.91 | 32.39 |
| Transformed Age | 5.31 | 1.49 | 5.95 | **<0.0001** | 3.06 | 9.21 |
| Transmission (High vs. Low) **x** Transformed Age | 0.59 | 0.21 | -1.51 | 0.131 | 0.29 | 1.17 |
| Transmission (High vs. Mod-High) **x** Transformed Age | 0.23 | 0.07 | -4.70 | **<0.0001** | 0.13 | 0.43 |
| Infection Status (Uninfected vs. Asymptomatic) | 0.20 | 0.06 | -5.09 | **<0.0001** | 0.11 | 0.38 |
| Transmission (High vs. Low) **x** Infection Status (Uninfected vs. Asymptomatic) | 2.44 | 0.45 | 4.85 | **<0.0001** | 1.70 | 3.49 |
| Transmission (High vs. Mod-High) **x** Infection Status (Uninfected vs. Asymptomatic) | 0.25 | 0.07 | -5.31 | **<0.0001** | 0.15 | 0.42 |
| Infection Status (Uninfected vs. Asymptomatic) **x** Transformed Age | 3.42 | 0.96 | 4.38 | **<0.0001** | 1.97 | 5.94 |
| Year | 0.95 | 0.01 | -5.80 | **<0.0001** | 0.93 | 0.97 |
| Infection Status (Uninfected vs. Asymptomatic) **x** Year | 1.12 | 0.02 | 5.37 | **<0.0001** | 1.08 | 1.17 |
| **Time Varying Covariates** | | | | | | |
| Infection Status (Uninfected vs. Asymptomatic) | 0.69 | 0.08 | -3.34 | **0.001** | 0.56 | 0.86 |
| Year | 0.94 | 0.01 | -7.29 | **<0.0001** | 0.92 | 0.95 |

A sensitivity analysis was carried to evaluate the effect of using pre-defined cut-offs of parasitemia for defining a febrile episode. The results were similar to that using one cut-off for parasitemia. The p-values in bold represent those that were statistically significant (p < 0.05). The symbol **x** indicates an interaction between the respective covariates. Abbreviations: mod-high - moderate-high.
