# Supplementary material for: Transmission and Age Impact the Risk of Developing Febrile Malaria in Children with Asymptomatic Plasmodium falciparum Parasitemia
Source: J Infect Dis. 2018 Oct 11;219(6):936–44. doi: 10.1093/infdis/jiy591 (PMC6386809; doi:10.1093/infdis/jiy591)
Supplement: jiy591_suppl_Supplementary_Material [file jiy591_suppl_supplementary_material.docx]

# Supplementary Material

## Supplementary Tables

The tables below show the detailed process of model optimization. Supplementary Table 1 shows the Schoenfeld’s residuals for the covariates tested and evaluates which ones varied over time. 2 shows the univariate analysis that evaluated the impact of varying parasitemia cut-offs (≥ 1, ≥ 2500, ≥ 10,000 parasites per microliter) on the risk of developing febrile malaria. Multivariate analysis to test the effect of different covariates on the risk of developing febrile malaria are presented in the following tables: Supplementary Table 1 shows odds ratio for all the covariates tested without time varying covariates and exponential time. Supplementary Table 4 is an advancement of Supplementary Table 3 and contains time-varying covariates as well and exponential time. Supplementary Table 5 is similar to Supplementary Table 4 but the covariate “sex” was dropped as it was found not to be significant. Supplementary Table 6 contains all the covariates that were found to be significant but with log-transformed time. Supplementary Table 7, 8 and 9 are similar to Supplementary Table 6 but with exponential time and alternative cut-off times of <30 & >30, <60 & >60 and <120 & >120, respectively. Supplementary Table 10 shows the markers of goodness-of-fit for the Cox-Regression Models tested above. Supplementary Table 11 shows the sensitivity analysis that tested the effect of all covariates and included different parasite density thresholds for defining clinical malaria as per previous work (i.e. any parasitemia in children < 1 year and ≥ 2500 parasites/µL for older children) per previous work (i.e. any parasitemia in children < 1 year and ≥ 2500 parasites/µL for older children) [1].

## References

1. Mwangi TW, Ross A, Snow RW, Marsh K. Case definitions of clinical malaria under different transmission conditions in Kilifi District, Kenya. J Infect Dis. 2005;191:1932–9.
