# Supplementary material for: Transmission and Age Impact the Risk of Developing Febrile Malaria in Children with Asymptomatic Plasmodium falciparum Parasitemia
Source: J Infect Dis. 2018 Oct 11;219(6):936–44. doi: 10.1093/infdis/jiy591 (PMC6386809; doi:10.1093/infdis/jiy591)
Supplement: jiy591_suppl_Supplementary_Figure [file jiy591_suppl_supplementary_figure.docx]

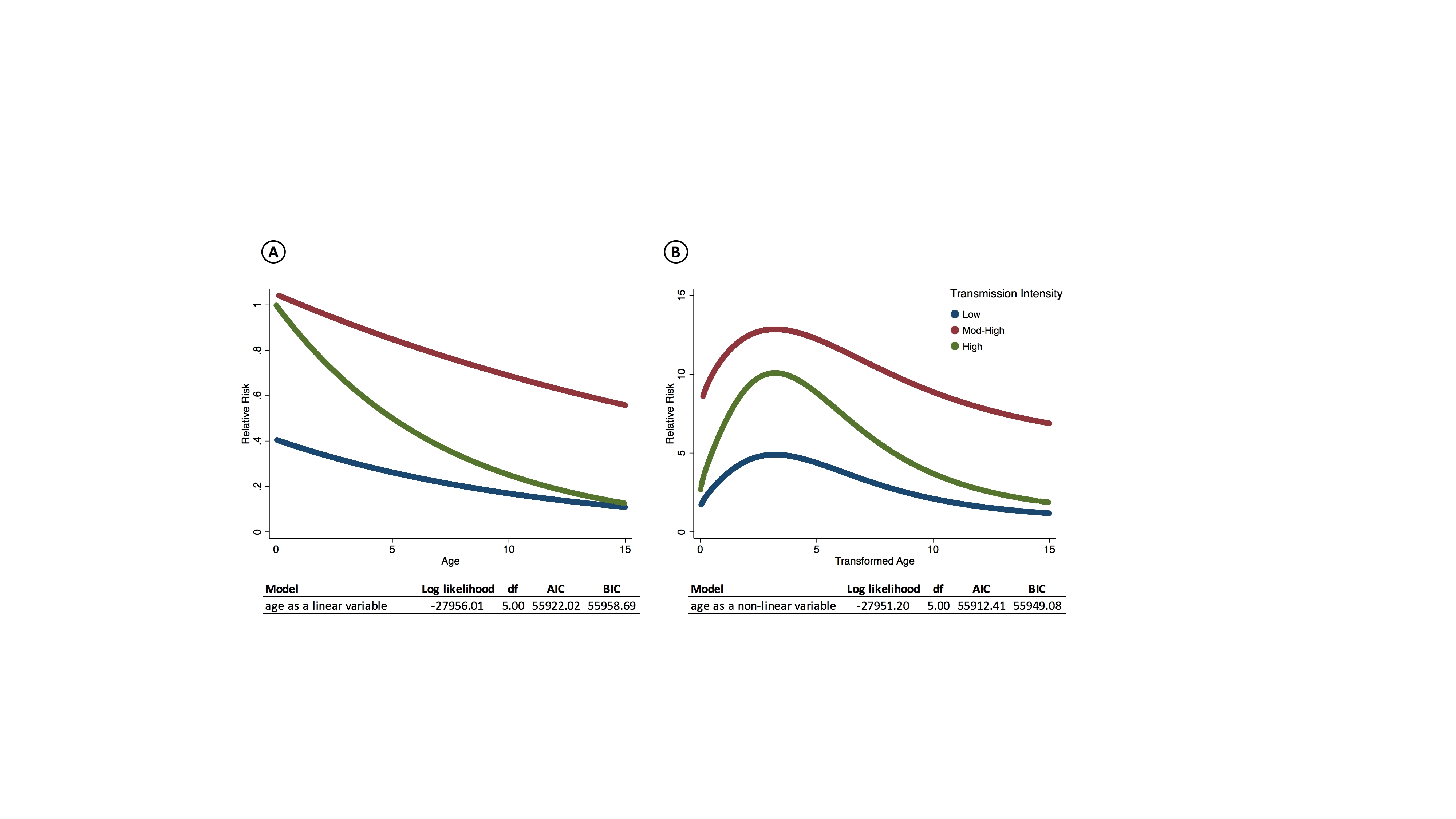


**Supplementary Figure 1. Models comparing the relative risk of febrile episodes as predicted from the interaction between site and age as (A) linear and (B) non-linear (transformed using fractional polynomials).** The Akaike's Information Criterion (AIC) and the Bayesian Information Criterion (BIC) below each respective graph show that the non-linear model is a better fit since the non-linear model has smaller log-likelihood values. For this reason, further models were constructed with non-linear age.
